# Research progress of chronic fatigue in neurodegenerative diseases

**DOI:** 10.1007/s10072-025-08254-z

**Published:** 2025-06-10

**Authors:** Luxuan Xia, Liye Wang, Wenyang Tian, Qianyu Zhao, Xinning Zhang, Shenghua Kang, Yao Wang, Xiaoxue Li, Wenxuan Guo, Jingmei Zhao, Yuanxin Zhang, Zhigang Chen, Nannan Li

**Affiliations:** 1 Beijing University of Chinese Medicine, 100029 Beijing, China; 2 Neurology Department One, Dongfang Hospital, Beijing University of Chinese Medicine, No. 6, Fangxingyuan Community, Fangzhuang, Fengtai District, Beijing, 100078 China; 3 Tsinghua University, Beijing, 100084 China; 4 Department of Acupuncture and moxibustion and Physiotherapy, The 305th Hospital of Chinese People’s Liberation Army, No. A-13, Wenjin Street, Xicheng District, Beijing, 100017 China

**Keywords:** Parkinson’s disease, Multiple system atrophy, Chronic fatigue, Research progress

## Abstract

The incidence of Neurodegenerative Diseases is on the rise, paralleling the societal trend of an aging population and the extension of the average life expectancy. NDs frequently manifest varying levels of chronic fatigue, a phenomenon whose underlying mechanisms remain obscure. This condition significantly impairs the quality of life for patients and presents a formidable challenge to our nation’s healthcare system. This paper delves into the potential mechanisms, assessments, and therapeutic approaches for Parkinson’s Disease and Multiple System Atrophy in the context of chronic fatigue. The objective of this study is to enhance the understanding of chronic fatigue in NDs, as well as to contribute to the advancement of its diagnosis and treatment methodologies.

Common neurodegenerative disorders(NDs) encompass a spectrum of conditions including, but not limited to, Alzheimer’s disease, Parkinson’s disease (PD), Multiple Sclerosis (MS), Multiple System Atrophy (MSA), dementia with Lewy bodies, and amyotrophic lateral sclerosis (ALS), among others. These conditions typically exhibit a gradual and progressive course of development. The World Health Organization forecasts that by the year 2040, neurodegenerative diseases will surpass cancer as the second most common cause of mortality globally. Patients afflicted with neurodegenerative disorders often exhibit varying degrees of chronic fatigue, which significantly detracts from their quality of life and impairs their social functioning. In the case of PD, the prevalence of fatigue is estimated to be around 50% [1], with nearly one-third of patients identifying fatigue as their most debilitating symptom [2]. A cross-sectional study has indicated that fatigue affects 29% of individuals with MSA [3].

The etiology of chronic fatigue in patients with NDs remains elusive [4]. Fatigue in PD may be associated with the interplay of various neurotransmitters, central and autonomic nervous system dysfunction, metabolic dysfunction, and other factors [5]. Research on MSA in conjunction with fatigue is scarce, with potential links to the dysfunction of the raphe nucleus and serotonin (5-HT) in the brainstem [6]. Current primary research directions for the treatment of chronic fatigue in NDs include pharmacotherapy, deep brain stimulation, exercise rehabilitation, and fatigue management strategies [7]. However, robust clinical evidence supporting these interventions remains limited. Traditional Chinese medicine (TCM) has demonstrated significant therapeutic advantages in the treatment of NDS, yet there has been a dearth of studies concentrating on the symptom of fatigue. This article provides a comprehensive review of the research advancements related to chronic fatigue in the context of PD and MSA as follows.

## Pathophysiology

### Potential pathophysiology of chronic fatigue in Parkinson’s disease

PD is a prevalent neurological condition among the elderly, with an estimated global prevalence of approximately 1% in individuals over the age of 60, and 3-5% in those over 70 [8]. The hallmark clinical features of PD include bradykinesia, muscle rigidity, and resting tremor, which are associated with the degeneration of the nigrostriatal dopaminergic pathway. Fatigue is a common non-motor symptom of PD, with a prevalence rate of 55% [9], and in some studies, it is as high as 70% [10]. Over half of PD patients consider fatigue to be one of the most debilitating symptoms of the disease [11]. The comparable prevalence of fatigue between newly diagnosed PD patients and those undergoing established therapeutic regimens indicates that current standard treatments fail to ameliorate this debilitating symptom [1]. Once present, fatigue symptoms may persist and can intensify over time as PD progresses [12, 13]. Reports from multidisciplinary expert symposiums suggest that fatigue in PD should be considered a syndrome rather than a single symptom [14], potentially linked to Chronic Fatigue Syndrome (CFS). Fatigue in PD is correlated with other non-motor symptoms, including apathy, depression, anxiety, excessive daytime sleepiness [1], memory decline [15], cognitive dysfunction, and autonomic dysfunction [16, 17]. A prospective cross-sectional prevalence study involving 246 patients indicated that subjective fatigue severity scores are associated with the progression of PD motor function, and these scores can predict motor function in PD patients [18]. Studies [19] have confirmed a possible relationship between fatigue and a high incidence of sleep disorders, with up to 50% of PD patients experiencing excessive daytime sleepiness [20]. The potential role of gender or age in the prevalence of PD-related fatigue has not been confirmed by research. Fatigue impacts patients’ hobbies, physical activities, and social engagements, reducing the quality of life throughout the disease course and causing psychological distress for PD patients.

Due to the complexity of fatigue, which involves both physiological and psychological aspects, its underlying mechanisms are not yet fully understood. Current research suggests that the potential pathophysiological mechanisms of fatigue in PD may include the interplay of various neurotransmitters, with the most significant likely being dopamine (DA), as well as serotonin and norepinephrine [21], central and autonomic nervous system dysfunction, metabolic dysfunction, and other hypotheses.

#### Related neurotransmitter

##### Dopaminergic pathway

It is widely recognized that the progression of PD is accompanied by the degeneration of dopaminergic neurons in the substantia nigra and striatum, with a positive correlation between the two. The relationship between DA and PD-related fatigue has not reached a consensus, but there is a tendency towards a correlation. In the Early L-DOPA Therapy in Early Parkinson’s Disease (ELLDOPA) cohort of untreated early PD patients, one-third of patients experienced a 50% improvement in fatigue after 42 weeks of levodopa treatment [22]. However, in the same ELLDOPA cohort, the relationship between striatal DA transporter and fatigue, studied using the novel dopamine transporter SPECT imaging agent ([123I]-β-CIT SPECT), was not statistically significant. A study [23] reported that striatal dopaminergic deficits are predictive factors for fatigue in mild PD patients. Additionally, rat studies [24] have shown that extracellular DA and 5-HT concentrations in the substantia nigra increase during exhaustive exercise-induced fatigue. Pharmacological evidence [21] also supports the important role of dopamine in central fatigue. Pavese [25] conducted PET scans on PD patients with and without fatigue, finding similar uptake of dopaminergic markers in most regions, but a significant reduction in the caudate nucleus and insula in the fatigue group. The authors suggest that dopaminergic dysfunction in the insula may play a role in the pathophysiology of PD-related fatigue. Some researchers believe [26] that DA deficiency may lead to cortical compensatory phenomena, with cortical excitability increasing and then decreasing as the disease progresses, thus increasing fatigue. Chen [27] found a significant correlation between fatigue and disease duration, severity of motor symptoms, suggesting that dopaminergic dysfunction may be involved in the pathogenesis of fatigue in PD patients. In summary, dopaminergic dysfunction in the striatum, caudate nucleus, and insula may be involved in the pathophysiological mechanisms of PD-associated fatigue.

##### Non-dopaminergic pathways

Mattia [1] propose that there is no correlation between fatigue and motor symptoms, indirectly supporting the hypothesis that non-dopaminergic pathways contribute to fatigue in PD. Research [28] indicates that the involvement of non-dopaminergic systems, such as the noradrenergic, cholinergic, monoaminergic, and serotonergic systems, may play a significant role in PD-related fatigue [29].

Pavese [25] conducted PET scans on PD patients with and without fatigue to verify whether fatigue is associated with serotonergic dysfunction at the level of the basal ganglia and limbic structures. Compared to PD patients without fatigue, those with fatigue exhibited a significant reduction in the binding of serotonergic markers in regions including the putamen, caudate nucleus, ventral striatum, thalamus, anterior cingulate, amygdala, and insula. The authors concluded that PD-related fatigue is associated with a decrease in serotonergic function within the basal ganglia and limbic structures. Zuo [30] found that PD patients with fatigue had lower concentrations of serotonergic metabolites in cerebrospinal fluid. Alterations in serotonergic signaling may lead to dysfunction of the frontal-basal ganglia circuitry and impaired integration of limbic inputs and motor functions, which could be a potential mechanism for PD fatigue [31]. PET study results in patients with CFS showed [32] impaired serotonergic transmission in cortical and subcortical regions, including the basal ganglia and limbic system, suggesting a possible link between PD fatigue and CFS. The correlation between fatigue and non-motor symptoms also supports this hypothesis. Depression [26], apathy, anxiety, somnolence [33] and sleep disorders [34] are all associated with low serotonergic pathways [35] and abnormal activity [36] in the limbic-cortical circuits.

Compared to healthy individuals, PD patients exhibit reduced levels of norepinephrine, which is associated with depression [37]. The deficiency of the cholinergic system is linked to cognitive dysfunction and dementia in PD [38].The close association of fatigue with non-motor symptoms of PD indirectly supports the possibility that noradrenergic and cholinergic pathways may play a role in PD-related fatigue. The uptake of striatal 11 C-dihydrotetrabenazine (DTBZ) is an important predictor of fatigue in patients with mild PD, suggesting the involvement of the monoaminergic pathway [23].

Therefore, the non-dopaminergic systems in the basal ganglia and limbic regions (especially low serotonin levels) and dopaminergic dysfunction in the striatum, caudate nucleus, and insula may play significant roles in PD-related fatigue. The basal ganglia participate in the limbic modulation of cortical motor output through the dopaminergic system and serotonergic pathways. This limbic modulation may affect the motivation for physical activity, thereby influencing the ability to maintain voluntary activation after exercise, leading to fatigue [39].

#### Related central nervous system

Specific lobar changes in the brain are likely to be the cause of fatigue in PD patients. A PET study [40] confirmed that brain regions involved in emotional, motor, and cognitive functions, such as the frontal, temporal, and parietal lobes (hypermetabolism in the right middle temporal gyrus and left middle occipital gyrus; hypometabolism in the right precuneus, left inferior frontal gyrus, and left superior frontal gyrus), are implicated in the fatigue mechanism of PD patients. Abe [41] used single-photon emission computed tomography (SPECT) to compare cerebral blood flow between PD patients and healthy controls, finding a significant correlation between fatigue in PD patients and reduced perfusion in the frontal lobe. A study [42] using regional homogeneity (ReHo) analysis of fMRI images found that fatigue in PD patients is related to the frontal regions, postcentral gyrus, and anterior cingulate cortex (ACC area) of the brain, which are involved in sensory, motor, and cognitive functions. Suk et al. compared the white matter fiber bundles of PD patients with and without fatigue and found that PD patients with fatigue had significantly higher mean fractional anisotropy (FA) values in all white matter bundles except for the left cingulum-hippocampus (CH), right superior longitudinal fasciculus, and right temporal longitudinal fasciculus; lower mean diffusivity (MD) values in all white matter bundles except for the left CH and uncinate fasciculus; and significantly reduced mean radial diffusivity (RD) values in white matter bundles except for the left CH. The study [43] suggests that changes in white matter tracts could serve as biomarkers for motor fatigue in PD. Kluger [44] found that PD fatigue is associated with specific atrophic changes in the caudate and putamen, which were not found in highly fatigued adults without PD matched for age, proving this to be a unique pathological mechanism for fatigue in PD patients.

Fatigue in PD is also associated with changes in neural signaling pathways. Lou [45] investigated the excitability of the primary motor cortex during and after exercise, with results suggesting that fatigue in PD is associated with abnormal excitability of the primary motor cortex. An increasing number of studies suggest that decision-making impairments are more common in PD patients and may lead to fatigue [46]. The mechanism may be the result of disrupted reciprocal circuits between the striatum and the orbitofrontal cortex (OFC) following dopamine depletion [47]. The OFC is involved in emotional regulation, reward processing, and decision-making [48]. Naia used the Iowa Gambling Task (IGT) to simulate real-life decision-making processes and, incorporating Damasio’s somatic marker hypothesis [49]—(the body’s emotion-based somatic signals during decision-making are integrated into higher brain regions to modulate future decision-making processes. Abnormal processing of somatic markers may lead to subjective fatigue.), observed that fatigued PD patients performed worse on the IGT, which is related to OFC function, compared to non-fatigued PD patients. The poor performance on the IGT was attributed to abnormal somatic markers. Tessitore [50] used resting-state functional magnetic resonance imaging (rs-fMRI) and demonstrated that untreated PD fatigue is associated with reduced connectivity in the supplementary motor area and increased connectivity in the default mode network involving the prefrontal and posterior cingulate cortices. Another hypothesis [51] posits that PD fatigue involves pathological processes spreading from the olfactory bulb to limbic regions. Mogenson [52] proposed that the basal ganglia filter signals from limbic structures and the cerebral cortex, thereby influencing other systems, and thus it can be speculated that changes in the filtering mechanism may lead to PD fatigue. Research on motor execution indicates that Parkinson’s Disease (PD) patients face difficulties in performing synchronized and sequential movements. During movement execution, PD patients experience a progressive slowing of movement speed or a decrease in amplitude as the sequence of movements continues, a phenomenon known as the sequential effect. Abnormal cortical plasticity and aberrant connectivity between the premotor cortex and the primary motor cortex can lead to the emergence of the sequential effect, which may be one of the mechanisms behind fatigue in PD patients. Although no studies have found a correlation between the sequential effect and fatigue [53], research based on this hypothesis may offer more possibilities.

#### Related autonomic nervous system

Autonomic dysfunction may be one of the pathophysiological aspects of PD-related fatigue. Ahn [16] conducted autonomic function tests on 89 untreated PD patients, confirming the conclusions of previous studies by Zuo [30] and Kelvin [19]. The study results indicated that 38.1% of patients with fatigue exhibited orthostatic hypotension (OH), compared to only 14.7% in the non-fatigue group. Additionally, the proportion of patients with clinical manifestations of OH in the fatigue group was significantly higher than in the non-fatigue group. The authors believe that there is a strong correlation between fatigue and autonomic dysfunction, particularly with OH.

Nakamura [54] reported a significant correlation between cardiac sympathetic denervation and fatigue in 33 PD patients, with fatigued patients often having a greater pressor response, indicating peripheral vascular denervation hypersensitivity. Cardiac sympathetic denervation may clinically manifest as an inability of the myocardium to increase contraction during exercise, leading to shortness of breath and thus inducing fatigue [55], or because fatigue shares the same pathophysiological mechanism with autonomic dysfunction, namely, the deposition of Lewy bodies in the locus coeruleus and the reticular formation [56].

#### Related metabolism

Inflammatory markers, such as C-reactive protein and cytokines, are potential biomarkers for fatigue in PD. Studies [57] have demonstrated a positive correlation between elevated levels of inflammatory markers and the degree of fatigue. Compared to PD patients without fatigue, those with fatigue exhibit higher levels of IL-6 [58] and higher levels of α-synuclein oligomers in cerebrospinal fluid, along with lower levels of amyloid β1–42 (Aβ1–42) [30]. Nikitina [59] used the Fatigue Severity Scale to assess the presence of fatigue in PD patients. The results indicated a strong correlation between the severity of fatigue and inflammatory markers such as CCL5, sVCAM-1, NCAM, and slCAM-1.An active inflammatory response may induce an increase in peripheral pro-inflammatory cytokines, which, after crossing the blood-brain barrier, can impede astrocytic reuptake of glutamate. The high extracellular levels of glutamate can affect neuronal activity and plasticity, thereby leading to fatigue [60, 61].

Chaudhuri [62] suggest that dysfunction of the hypothalamic-pituitary-adrenal (HPA) axis is associated with fatigue in PD. Kluger [63] also believe that fatigue is closely related to endocrine dysfunction or circadian rhythm disorders in the hypothalamus. Kenangil [64] and Okun [65] have studied the relationship between testosterone levels and fatigue in male PD patients. Although PD patients have lower levels of free testosterone compared to the control group, there is no correlation with fatigue severity scores, and the impact of testosterone treatment on fatigue in PD patients is not statistically significant.

### Potential pathophysiology of chronic fatigue in multiple system atrophy

MSA is a progressively debilitating neurodegenerative disease with clinical manifestations that include autonomic dysfunction, a Parkinsonian syndrome with poor response to levodopa therapy, cerebellar ataxia, and pyramidal signs, among other symptoms. The prevalence of MSA ranges from 0.5 to 6.2 per 100,000 in European countries and 7.6–17 per 100,000 in non-European countries [66]. The average survival period from symptom onset is approximately 5–6 years [67]. The etiology of MSA remains unknown, and there is currently no effective treatment available. MSA can be classified into two subtypes based on the predominant motor symptoms: the Parkinsonian type (MSA-p) and the cerebellar type (MSA-c). Non-motor symptoms of MSA include fatigue, depression, anxiety, sleep disturbances, and pain. Fatigue is a common symptom in patients with MSA, significantly reducing their sense of social participation and quality of life [68].

#### Epidemiological characterization of fatigue prevalence in MSA patients

Due to the lack of a standardized definition and measurement method for MSA-related fatigue, different studies have varying definitions and screening scales for fatigue, leading to discrepancies in the reported prevalence of fatigue among MSA patients. A cross-sectional study involving 174 Chinese MSA patients reported a fatigue prevalence of 28.7% [3]; another American study [69] investigating the initial symptoms of MSA patients showed a post-exertional fatigue rate of 39% (7 out of 18); a Swedish study [70] using the Chronic Disease Treatment Assessment Scale reported a fatigue prevalence of 78.5% (11 out of 14) in MSA patients, and an Italian study [71] indicated a fatigue prevalence of 82.4% (28 out of 34). A prospective cohort study [72] followed 146 MSA patients for one year, and the results showed that 48.6% of patients experienced persistent fatigue, 11.6% had non-persistent fatigue, 15.1% never experienced fatigue, and 24.7% developed new-onset fatigue.

#### Correlates of fatigue in MSA

Zhang [72] conducted a one-year prospective study and found that the severity and frequency of fatigue in MSA increase with disease progression. The presence of OH was associated with fatigue in MSA-P (Parkinsonian subtype of MSA), while anxiety was associated with fatigue in MSA-C (Cerebellar subtype of MSA). A positive correlation was observed between younger age and fatigue in MSA, possibly due to the higher stress levels and susceptibility to tension in younger individuals, which may induce fatigue. However, Xu [3] confirmed that the prevalence of fatigue in MSA is not related to disease duration, OH, or the severity of motor symptoms; fatigue does not evolve with the progression of the disease, existing in the early stages and tending to persist for a period. Female gender, anxiety, Excessive Daytime Sleepiness (EDS), and the use of sleep medications may be associated with an increased risk of fatigue in MSA, with no statistically significant difference in the frequency of fatigue between MSA-P and MSA-C subtypes. Among patients with MSA and fatigue, the prevalence of anxiety was 54%. Multivariate analysis results showed that there is a correlation between anxiety and both fatigue and EDS, with an interactive effect present.

#### Potential mechanisms of fatigue in patients with multiple system atrophy

Meyer [6] demonstrated through [18 F]-MPPF PET that dysfunction of the 5-HT system in the dorsal raphe nucleus and brainstem may lead to fatigue in MSA. 5-HT neurons, located in the dorsal raphe nucleus, project signals to various regions of the brain, including the basal ganglia and limbic structures. Significant loss of 5-HT neurons in the dorsal raphe nucleus and other brainstem nuclei has been observed in MSA patients, along with reduced levels of serotonin metabolites in cerebrospinal fluid. Moreover, reduced 5-HT function has been identified as a potential mechanism for fatigue in PD and other neurological disorders, which may also be implicated in MSA-related fatigue. Studies have confirmed that neurofilament light chain (NFL), α-synuclein, glial fibrillary acidic protein (GFAP), brain-derived neurotrophic factor (BDNF), and triggering receptor expressed on myeloid cells-2 (TREM2) do not show significant correlation with fatigue and its progression in MSA. NFL is a sensitive marker of axonal injury, which increases in cerebrospinal fluid and blood with axonal damage. α-Synuclein is a major component of glial cytoplasmic inclusions in MSA patients. GFAP and TREM2 may be involved in the neuroinflammatory process of MSA, and BDNF plays a neurotrophic role in MSA patients. Future research on the mechanisms of fatigue in MSA patients should continue to explore other directions.

## Relevant assessment methods

Fatigue measurement exhibits a high degree of diversity, primarily due to the lack of a universally accepted definition and classification of fatigue, as well as varying diagnostic methods, which makes its assessment more challenging. Fatigue is a common and independent non-motor symptom that appears early in PD and MSA and may persist throughout the entire disease course; establishing a unified diagnostic criterion for disease-related fatigue is crucial. The most commonly used assessment method in current clinical practice and research is scales; in addition, various physical and cognitive measurement tools are actively being developed to better serve clinical and scientific research needs.

### Scale assessment

The pathophysiology of fatigue in NDs is not yet clear, and no recognized biological markers have been established. Although scales and questionnaires are subjective, they remain the primary tools for screening, measuring, and comparing fatigue in research studies. Commonly used questionnaires for fatigue in MSA and PD include: the Fatigue Severity Scale (FSS), the Multidimensional Fatigue Inventory (MFI), the Parkinson Fatigue Scale (PFS), the Fatigue Impact Scale for Daily(D-FIS), the Modified Fatigue Impact Scale(MFIS), the fatigue assessment instrument (FAI), the Functional Assessment of Chronic Illness Therapy-Fatigue (FACIT-F), the Bell CFIDS Disability Scale, the Chalder Fatigue Scale, the DePaul Pediatric Health Questionnaire, the DePaul Post-Exertional Malaise Questionnaire, the DePaul Symptom Questionnaire, the Nottingham Health Profile Energy Subscale (NHPEN), and the Fatigue Questionnaire (FQ), among others. The Movement Disorder Society (MDS) commissioned Friedman [73] to evaluate the application of existing clinical fatigue rating scales in PD: the FSS was recommended for screening and severity rating; the MFI was suggested for screening and recommended for severity rating; the PFS and FACIT-F were recommended for screening and suggested for severity rating.

Given the distinct fatigue manifestations and divergent pathophysiological mechanisms across different disease entities, no single scale can adequately capture the fatigue characteristics across all disease entities. Therefore, based on current understanding of fatigue associated with PD and MSA, the development of individualized scales urgently requires systematic initiation [74].

#### FSS

In 1989, Krupp [75] began to develop the FSS in order to standardize the measurement of fatigue severity in clinical patients. The FSS is a single - dimension self - assessment tool that contains 9 items. The scoring criteria are based on a 7 - point Likert scale (from very dissatisfied to very satisfied), with a score range from 9 to 63. A score of ≥ 36 indicates the presence of fatigue, and the higher the score, the more severe the fatigue. The FSS covers the impact of fatigue on various aspects of daily life, such as physical function, work ability, and social activities. It is the most commonly used fatigue scale f across all diseases and is the only scale recommended by the MDS task force for screening fatigue in PD and for severity rating. The FSS questionnaire is short in length and high in accuracy, and its Portuguese and Turkish versions have been proven to be effective for assessment. The FSS has been shown to have a high degree of internal consistency in PD patients [76], and this scale is also frequently used in fatigue research on MSA [77].

#### MFI

The MFI was developed by Professor Smets in 1995. It consists of 20 items and contains 5 dimensions, namely general fatigue, physical fatigue, mental fatigue, reduced activity, and decreased motivation. Different diseases may focus on different dimensions. Each dimension contains 4 items and adopts a 5 - point Likert scale, with each item scored from 1 to 5. If a single dimension score is ≥ 4 points and the overall score is ≥ 20 points, it indicates the presence of fatigue. The higher the score, the more severe the fatigue. Compared with the FSS, the MFI is more sensitive to the changes in fatigue levels before and after intervention [78]. Chen [76] confirmed and recommended using the MFI to screen the severity of mental fatigue in PD patients. The MFI has good internal consistency and inter - rater reliability. Its reliability and construct validity have been verified in PD patients [79]. Lou believed that using the MFI as a multi - dimensional questionnaire to assess the fatigue level of PD patients provides more flexibility, enabling researchers to distinguish and quantify physical and mental fatigue.

#### PFS

In 2005, Brown and colleagues developed a simple and easy-to-complete scale called the Parkinson Fatigue Scale (PFS) [80], specifically designed for patients with PD, and established its validity and reliability. The PFS [81] consists of 16 items rated on a 5-point scale (from very dissatisfied to very satisfied), asking patients to score their subjective experiences related to fatigue, and intentionally excludes the emotional and cognitive effects of fatigue to avoid overlap with other neurological manifestations in PD. The PFS is suitable for researchers and healthcare professionals to assess the degree of fatigue in PD patients and is routinely used in clinical practice and scientific research. Additionally, the PFS is available in multiple language versions, including Brazilian, Swedish, Chinese [27], Greek, and Turkish [82] editions, all of which have been validated for reliability and validity.

#### D-FIS、MFIS

The D-FIS was developed based on the Fatigue Impact Scale (FIS), which was originally created in Canada in 1994. The D-FIS is an 8-item self-report questionnaire with each item having 5 response options (ranging from 0 = no problem to 4 = extreme problem). The D-FIS score is the sum of the ordinal scores of each item. The Movement Disorder Society (MDS) classifies it as a “recommended” scale for assessing fatigue in Parkinson’s Disease (PD). The D-FIS has been demonstrated to possess good metric properties [83].

The MFIS, as an enhanced version of the original Fatigue Impact Scale (FIS), demonstrates improved internal consistency (Cronbach’s α > 0.9). This 21-item instrument employs a 5-point Likert scale (ranging from never to always) to evaluate the impact of fatigue across functional domains, while providing domain-specific composite scores for physical, cognitive, and psychosocial dimensions. Although the MFIS was not included in the MDS task force’s publication, its validity has been confirmed by Schiehser in Parkinson’s disease and is recommended by the Multiple Sclerosis Council’s clinical practice guidelines [84].Based on its performance in clinical trials, the MFIS may be more sensitive to changes in fatigue than some other available scales [85].

The D-FIS is designed to capture short-term dynamic fluctuations of fatigue severity, reflecting real-time variations in patients’ fatigue levels across different time points during daily activities. In contrast, the MFIS is utilized to evaluate the global impact of chronic long-term fatigue on daily functioning, with particular emphasis on assessing the comprehensive effects of fatigue on patients’ overall quality of life.

#### FAI

The FAI was developed by Joseph E. Schwartz from the Laboratory of Psychiatric Research and Lina Jandorf from the Laboratory of Neurology in the United States in 1993. The FAI is a multidimensional scale that can assess the severity of fatigue, the impact of the environment on fatigue, the consequences of fatigue, and factors that exacerbate or alleviate fatigue. The FAI focuses on the fatigue symptoms experienced by patients over the 2 weeks prior to assessment, comprising 29 items. Each item is rated on a 7-point scale ranging from “completely disagree” to “completely agree,” with higher scores indicating greater fatigue severity [78]. The FAI not only provides a precise quantification of fatigue but also reflects individualized fatigue characteristics and identifies factors influencing fatigue, with results that are intuitive and comprehensive [86]. Compared to scales such as the MFI and FSS, the FAI has unique advantages in assessing the environmental specificity of fatigue and its response to rest and sleep, allowing for a more comprehensive coverage of different dimensions and manifestations of fatigue. In the context of PD, there is typically a lack of systematic evaluation of the reliability and validity of the FAI.

#### FACIT-F

The FACIT-F is part of the FACIT (Functional Assessment of Chronic Illness Therapy) series of scales. It was developed in the 1990s by David Cella and colleagues at Northwestern University in the United States, aiming to assess the quality of life and symptom burden of patients with chronic diseases. The scale contains 13 items covering multiple dimensions, including physical, psychological, and social functions. It is more sensitive and suitable for longitudinal studies to detect changes in symptoms in a timely manner [87, 88].

### Non-scale-based assessment

#### Physical fatigue quantification

Finger tapping is a motor task commonly used to assess the severity of fatigue in PD. Lou’s laboratory [26] utilizes an electronic keyboard equipped with Musical Instrument Digital Interface (MIDI) technology to study the degree of fatigue in PD patients’ finger tapping. In this task, subjects are asked to alternately press two keys placed 20 centimeters apart as quickly as possible for 30 s. A computer records the timing and duration of each key press. Physical fatigue is measured by the decline in instantaneous tapping frequency, dwell time (the duration the finger remains on a key), or flight time (the time from releasing one key to starting to press another key) within 30 s.

In the laboratory [39], continuous and intermittent maximal voluntary contraction (MVC) training protocols are commonly used to assess physical fatigue. The total amount of fatigue can be quantified through the fatigue index, which is the ratio of the maximum voluntary contraction force before and after the induction of fatiguing exercise. Peripheral fatigue is typically measured by the decrease in muscle twitch force induced by electrical stimulation of peripheral nerves, while central fatigue is quantified by the decrease in activation level of the maximum voluntary muscle contraction following fatiguing exercise.

#### Mental fatigue quantification

Oken [89] utilized reaction time (RT) to detect attention, while Lou [26] quantitatively assessed mental fatigue by measuring RT or error rates over a period of time. An increase in RT or error rates over time indicates the presence of mental fatigue.

Given that PD patients have deficits in three neurotransmitter systems [90] (noradrenergic, cholinergic, and dopaminergic), Lou [26] also employed the Attention Network Test (ANT) to examine mental fatigue in PD patients, with the testing session lasting over 51 min. The study results suggest that the ANT is a useful tool for quantifying mental fatigue in PD patients.

## Therapeutic advances

### Western medical treatment

The first and most crucial step in treating fatigue in NDs patients is to explain to the patients and their families that it is an important and common symptom of NDs. They must also recognize that, due to the undetermined underlying pathophysiological mechanisms, there is currently no established treatment method. At present, attempted treatment options for PD-related fatigue include pharmacological therapy, deep brain stimulation, exercise rehabilitation training, and fatigue management. there is limited understanding of fatigue in MSA, and its mechanisms remain largely enigmatic. Consequently, the treatment of MSA-related fatigue is highly challenging, and there is no widely accepted therapeutic regimen in the industry. Current approaches are mostly derived from treatment experiences with PD-related fatigue, including pharmacological treatment, DBS, and rehabilitation therapy.

#### Western Pharmacological therapy

Currently, there is no robust evidence to demonstrate that pharmacological treatment can improve fatigue in PD. Levodopa, modafinil, methylphenidate, and rasagiline may potentially be effective for PD-related fatigue.The pharmacological research on MSA-related fatigue has not yet been initiated, and in clinical practice, fatigue is often indirectly alleviated by improving other symptoms of MSA. This article only covers the pharmacological research related to PD fatigue.

##### Levodopa

Lou [45] conducted a double-blind, placebo-controlled, crossover study on 25 PD patients, which demonstrated that when PD patients were in the “off” state, levodopa could reduce physical fatigue in finger-tapping exercises, normalizing the excitability of cortical-motor neurons before, during, and after movement in PD patients. Schifitto [22] carried out a randomized, double-blind, placebo-controlled clinical trial involving 349 patients, indicating that the group of PD patients treated with levodopa experienced less progression of fatigue.

##### Stimulants

Lou [26] randomly assigned 19 PD patients to modafinil and placebo groups in a double-blind manner for a period of two months. The results indicated that modafinil could reduce fatigue in PD patients under the conventional PD treatment regimen. A randomized controlled trial [91] demonstrated that methylphenidate (commonly known as Ritalin) could improve fatigue scores in PD patients. In this study, 36 patients were randomly divided into methylphenidate and placebo groups for a period of six weeks. The results showed significant improvements in FSS and MFI for the methylphenidate group, whereas there were no improvements in the placebo group.

**Rasagiline**: Stocchi [92] conducted a double-blind, randomized clinical trial on 1105 untreated PD patients, dividing them into three groups: one receiving 1 mg of rasagiline daily (*n* = 270), another receiving 2 mg daily (*n* = 277), and a placebo group (*n* = 558), over a period of 9 months. Compared to the placebo group, the progression of fatigue was significantly reduced in those receiving 1 mg or 2 mg of rasagiline daily. Lim [93] assessed the impact of rasagiline on fatigue in PD patients and found that, at the end of 12 weeks, patients taking 1 mg of rasagiline daily exhibited lower levels of fatigue compared to the placebo group.

##### Dopaminergic agonists

The effect of dopaminergic agonists on fatigue is still uncertain, and fatigue may even be a side effect of these drugs [94]. Ongre [95] suggests that untreated PD patients who have been treated with dopaminergic agonists for one year experience a reduction in fatigue, with greater benefits compared to the use of levodopa.

##### Duloxetine

Duloxetine is a novel selective serotonin-norepinephrine reuptake inhibitor (SNRI). It can improve fatigue in patients with PD by regulating the levels of 5-HT and NE, independent of its effect on depressive symptoms [96]. The combination of duloxetine with anti-PD medications in an optimized treatment plan not only ameliorates PD-related fatigue but also alleviates other motor and non-motor symptoms, contributing to the enhancement of patients’ quality of life.

#### Western Non-Pharmacological therapy

##### Associated with PD: Deep Brain Stimulation

Bilateral subthalamic nucleus deep brain stimulation (DBS) has shown good efficacy in improving motor function in PD patients, but its effects on fatigue have been little studied.Some research results indicate [97] that PD patients who have undergone bilateral subthalamic nucleus DBS do not exhibit significant changes in the severity of fatigue. However, another study found [98] that fatigue is commonly reported after DBS in PD patients and can negatively impact their quality of life.

##### Exercise therapy

It is well recognized that exercise can improve cardiovascular and cerebrovascular function, reduce the incidence of osteoporosis, and enhance psychological well-being. Provided that compliance is achieved, moderate, individualized exercise training has few side effects and can be widely applied. Kelly [99] guided 15 PD patients through a 16-week program of high-intensity individualized exercise training, integrating strength, endurance, and balance components. The FSS scores indicated a significant improvement in the patients’ fatigue levels, which was associated with increased muscle fiber hypertrophy and enhanced mitochondrial complex activity. Although this study had a small sample size of only 15, it fundamentally demonstrated that high-intensity individualized exercise can ameliorate fatigue. A small randomized controlled trial confirmed [100] that home treadmill training can reduce fatigue in patients with mild PD. However, another preliminary study suggested [101] that a community exercise program did not improve fatigue, possibly due to low patient compliance in that study. Cugusi [102] investigated the effects of adaptive physical activity on motor and non-motor symptoms in PD patients. Nine PD patients underwent a regimen three times a week for nine weeks. The results showed a significant improvement in PFS scores, as well as enhancements in patients’ motor function and quality of life. In a study [103] involving 60 PD patients on the effects of aerobic walking on fatigue, fatigue (measured by FSS) was significantly improved. Current evidence tends to support a positive role of exercise in combating PD-related fatigue, although patient compliance remains a significant factor affecting the success of this therapeutic approach.

##### Fatigue management

Initially conducted in face-to-face group settings, fatigue management has been adapted to various formats, including telephone conferences, online internet platforms, and one-on-one sessions. The program [104] guides energy management strategies such as activity simplification, task analysis, environmental modifications, and prioritized planning. Fatigue management has been evaluated in several countries, including the United States, Australia, and the Netherlands. However, its impact on PD patients remains unknown. Neda Alizadeh [105] will explore the effects of fatigue management on PD patients’ fatigue improvement through a randomized controlled trial in the form of video conferencing, assessing its feasibility in PD patients. Should this program prove beneficial to patients, this study will provide preliminary evidence to support further refinement of the program.

##### Associated with MSA

Among these, repetitive Transcranial Magnetic Stimulation (rTMS) has shown significant effects [106]. Previous studies [107] have indicated that rTMS applied to the left dorsal lateral prefrontal cortex (DLPFC) can help improve fatigue symptoms in neurological disorders such as fibromyalgia, ME and MS. The optimal frequency for rTMS treatment of fatigue is 10 Hz, with an evidence grade of a. A single-center, randomized, double-blind, controlled trial [106] involving 22 MSA patients with fatigue measured the FSS-9, UMSARS, HAMD, and HAMA scale scores at baseline, and at 1 week, 2 weeks, and 4 weeks post-treatment, confirming that rTMS applied to the left DLPFC can provide short-term relief for fatigue in MSA patients. However, the beneficial effects do not last longer than 4 weeks. The short duration of beneficial effects may be related to the continuous progression of MSA symptoms. High-frequency rTMS can increase the cortical excitability of the DLPFC, promote dopamine release, and reduce the transmission of inhibitory neurotransmitters such as GABA, thereby having a beneficial impact on fatigue symptoms.

It is unclear whether MSA fatigue shares common pathophysiological mechanisms with other related factors, and further research is warranted to determine if treating associated factors such as daytime sleepiness and anxiety can alleviate fatigue in MSA patients. Similarly, whether exercise, rehabilitation, and fatigue management can mitigate fatigue associated with MSA should also be the subject of further investigation.

### Traditional Chinese medicine treatment

#### Associated with PD

The elements of Traditional Chinese Medicine (TCM) syndrome differentiation for PD-related fatigue are primarily based on “decreased marrow” [108], with compound syndrome elements [109] mainly involving “spleen deficiency + blood deficiency,” “spleen deficiency + decreased marrow,” and “kidney deficiency + yin deficiency.” Ginseng and Spleen-Returning Decoction [110] can improve the fatigue in elderly PD patients with qi-blood deficiency syndrome, while other syndrome types and basic formulas require further research. A study [111] with a sample size of 120 confirmed that the combination of acupuncture and conventional Western medical treatment for PD fatigue is effective, significantly improving patients’ fatigue symptoms and quality of life. In the early stage [112], where “internal stirring of liver wind” is predominant, treatment should calm the liver and regulate the spleen, using modifications of the Tremor Three Needles (Si Shen Needles, Si Guan, and Feng Chi points); in the middle stage, where “deficiency of both heart and spleen” is predominant, treatment should regulate the spirit and strengthen the spleen, using modifications of the Spirit-Regulating Needle Method (such as Si Shen Needles, Shen Ting, Yin Tang, Shen Men, San Yin Jiao, Zu San Li, Tai Chong, etc.); in the late stage, where “kidney yang deficiency” is predominant, treatment should unblock the Governing Vessel and tonify yang, using modifications of the Governing Vessel meridian points.Qigong exercises like “Yi Jin Jing” [113] can effectively alleviate fatigue symptoms in patients with mild to moderate Parkinson’s disease, improve their non-motor symptoms, and enhance their cardiopulmonary endurance and tolerance to exercise fatigue, thereby improving quality of life. A study by Ji Xianyue [114] has shown that Baduanjin (Eight Pieces of Brocade), the Mediterranean diet, and the combination of Five-Element music with acupuncture can improve fatigue in PD patients and reduce the PFS scores.

#### Associated with MSA

The pathophysiological mechanism of MSA [115] is generally considered to involve deficiencies of the liver, kidneys, and spleen, and dysfunction of qi, blood, and dampness transformation; clinical TCM treatments often focus on syndromes of liver-kidney deficiency, spleen-kidney deficiency, and phlegm-stasis obstruction, but research targeting fatigue symptoms urgently needs to be perfected. Wang Yao [116] believes that the pathogenesis of MSA is characterized by deficiency of the liver and kidney, and damage to the marrow due to impaired collaterals. After six months of treatment with a warming kidney and brain-strengthening formula, the FSS scores of patients significantly decreased (*P* < 0.05 for all).

## Summary and prospects

Fatigue occurs across various domains and its concept is often confused. Therefore, some scholars suggest that fatigue in PD should be studied as a syndrome rather than a symptom. Chronic Fatigue Syndrome (CFS) is a complex of persistent or relapsing fatigue as the primary manifestation, accompanied by a variety of neurological and psychiatric symptoms, without any organic or mental diseases [11]. By integrating the concept of CFS, we can regard fatigue in NDs as a sense of exhaustion that cannot be explained by the effects of medication or other mental disorders, existing within a specified time frame and associated with other fatigue-related symptoms, such as reduced motivation and non-restorative rest. Viewing NDs-related fatigue as a syndrome can enhance the comparability between studies and also expand the recruitment scope for research.

In today’s rapidly developing society, with the advent of an aging population, the prevalence of NDs is increasing year by year, and the symptom of fatigue cannot be ignored. When abnormally severe, this symptom can directly limit patients’ lives independently of other symptoms of NDs that may cause activity disorders. The potential pathophysiological mechanisms of PD-related fatigue include the collaboration of various neurotransmitters, related white matter lesions, autonomic dysfunction, and endocrine disorders. Currently, there is relatively little research on fatigue in MSA, which may be associated with the dysfunction of 5-HT in the dorsal raphe nucleus and brainstem.

At present, fatigue lacks a universally accepted definition and classification, making its measurement challenging: there are no straightforward physical quantitative measurement methods, and the variety of scales available lack clarity in specificity and universality. In the future, a substantial amount of research will be needed to provide updated recommendations and to distinguish the specificities of various scales, as well as to determine the screening, diagnostic, and evaluation scales for fatigue in different NDs. Of course, it is our hope that quantitative physical assessments, such as “finger tapping,” can gain consensus among experts, facilitating future clinical and scientific research endeavors.

There are currently no evidence-based guidelines for the treatment of fatigue in NDs. Pharmacological treatment, deep brain stimulation, exercise rehabilitation training, and fatigue management are the main areas of current research. Exercise rehabilitation training has good effects but requires ensuring patient compliance; more robust evidence is needed for pharmacological treatment, deep brain stimulation, and fatigue management. Future research should focus on identifying biomarkers and correlations of fatigue, which may provide direction for the development of effective treatment methods.

The workload for future research on fatigue in NDs is immense. It is imperative that we swiftly clarify diagnostic criteria and develop universal research methodologies based on these diseases. Establishing clinical guidelines that encompass diagnostic standards, pathophysiological mechanisms, clinical phenotypes, auxiliary examinations, assessment tools, and standardized questionnaires is crucial. Further research into biomarkers and viable targeted treatment methods is essential. Traditional Chinese medicine (TCM) has proven to be highly effective and safe in treating fatigue in NDs, without addiction or drug resistance, and with minimal adverse effects. It can fundamentally regulate patients’ constitution and effectively halt disease progression. Further research into the characteristics of TCM syndrome differentiation for fatigue in NDs could potentially bring hope to many patients and their families.

## Data Availability

The data analyzed in this review article are from previously published studies, which are cited within the manuscript. There are no newly generated data to declare.
